# Effect of Intraoral Drainage after Impacted Mandibular Third Molar Extraction on Non-Infectious Postoperative Complications

**DOI:** 10.3390/jcm10204705

**Published:** 2021-10-14

**Authors:** Grzegorz Trybek, Joanna Jarzęcka, Olga Preuss, Aleksandra Jaroń

**Affiliations:** Department of Oral Surgery, Pomeranian Medical University in Szczecin, 72 Powstańców Wlkp. St., 70-111 Szczecin, Poland; joanna.jarzecka@pum.edu.pl (J.J.); olga.preuss@pum.edu.pl (O.P.); jaronola@gmail.com (A.J.)

**Keywords:** third molar extraction, impacted third molar, third molar removal, mandibular third molar, complications, drainage, intraoral drainage, latex flat drain, flat drain, sodium–calcium alginate flat drain

## Abstract

Surgical removal of mandibular third molars is associated with non-infectious postoperative complications, including pain, swelling, trismus. Intraoral drains are noteworthy because of their ease of application, availability, and effectiveness. This study aimed to evaluate the use of latex and calcium–sodium alginate mouth flat drains on the severity of postoperative complications such as pain, trismus, and edema after surgical removal of mandibular third molars. Ninety patients who underwent surgical removal of wisdom teeth were studied. The patients were divided into three groups. Group A—with a latex flat drain, group B—with a sodium–calcium alginate (Kaltostat) flat drain, and group C—with a wound closed with knotted sutures. Patients were assessed for pain on a VAS scale every day from surgery to postoperative day 7. Before the procedure and on postoperative days 1, 2, and 7, the pain level, edema, and trismus were measured, respectively. Intraoral drainage with a flat drain after mandibular third molar removal does not significantly reduce pain, as measured by the VAS scale, or postoperative swelling, as measured by lines between craniometric points. Intraoral drainage with a latex drain after mandibular third molar removal does not significantly reduce trismus, while intraoral drainage with a calcium–sodium alginate drainage bag significantly increases trismus.

## 1. Introduction

Surgical removal of impacted mandibular third molars is a common procedure performed in oral surgery practices, accounting for a significant percentage of all procedures performed by oral surgeons [1]. Wisdom tooth impaction is a common source of the pain associated with eruption difficulties and can also cause periapical inflammation, abscesses, phlegmon, and cysts [2,3,4,5]. The surgical removal of the lower wisdom tooth is associated with the development of many complications. We can divide these complications into infectious and non-infectious [6]. Infectious complications include purulent alveolitis, abscesses, while non-infectious complications include trismus, pain, swelling, postoperative bleeding, post-extraction alveolitis (PAE), or alveolar osteitis (AO), also known as dry socket [7,8]. Proper tissue healing after surgical removal of wisdom teeth, in addition to general patient-dependent factors, is influenced by appropriate post-extraction wound care and surgical technique [9,10,11,12]. Intraoral drainage after surgical removal of a wisdom tooth can be accomplished using round or flat drains inserted into a release incision on the vestibular side of the mouth or top of the alveolar process of the mandible. The first to use the phrase “buccal drainage” (buccal drainage) in the title of their paper were Hu et al. in 2017, thus defining the location of a latex teat in the floor of the oral vestibule, on the buccal side, designed to drain buccal swelling and reduce buccal volume after surgical removal of the lower wisdom tooth [13]. Drainage after lower third molar extraction has been the subject of many studies and scientific publications. It affects the reduction of discomfort associated with the procedure; however, the results are inconclusive. Different types of incisions used in surgery during wisdom tooth extraction, sites of drain insertion, and materials used to drain secretions have been studied. Latex drainage is routinely used after incision of abscesses, causing the evacuation of the purulent contents, preventing tissue approximation and resealing. However, leaving the drain in place for 72 h results in wound epithelialization and the initiation of prolonged secondary healing [14]. The use of a drain after removing the third molar in the mandible is intended to create an outflow route for excess exudate fluid and minimize the risk of postoperative hematoma infection. It prevents its formation by gravitational drainage of the exudate formed in the first phase of tissue healing. This study aimed to find an optimal postoperative wound supply that would be the least discomforting to the patient and widely available. Conflicting reports on minimizing three parameters, i.e., pain, swelling, and trismus, with the use of a drain after surgical extraction of the third molar in the mandible inspired a study to answer the question: What parameters are affected by the use of a drain inserted in the floor of the oral vestibule after surgical treatment?

## 2. Materials and Methods

The study was conducted after receiving a positive opinion from the Bioethics Committee of the Medical University, No. KB-0012/38/18 on 12 March 2018.

### 2.1. Study Group

The study group consisted of 90 patients enrolled by convenience sampling of the Department of Oral Surgery of the Medical University who qualified for the planned removal of mandibular third molars. The study included adult patients, generally healthy non-smokers with no local or general inflammation symptoms. Exclusion criteria were: patients under 18 years of age, pregnancy and lactation, systemic diseases, clinical signs of pericoronal inflammation due to the difficult eruption of the eighth tooth, smoking, and allergy to latex. Before the procedure, patients were asked to complete a written consent for the procedure and participate in the research project. Patients were put into three groups by simple randomization:

- A (*n* = 30)—group with latex drain used;
- B (*n* = 30)—group with a sodium–calcium alginate drain applied;
- C (*n* = 30)—control group, in which the wound was secured with standard knotted sutures.

Simple randomization took place before the scheduled surgery; each patient received a sealed envelope with allocation to each group. Before the surgical removal of the lower wisdom tooth, the mandibular third molar’s degree of difficulty, morphology, and position were assessed based on a pantographic image. The classification according to Pell and Gregory and the surgery difficulty index according to Pederson were used for this purpose.

### 2.2. Surgery

Before surgical removal of the retained third molar in the mandible, each patient received 600 mg of clindamycin in a “one-shot” scheme. The surgical removal of the wisdom tooth was performed by one highly experienced operator with the same repeatable surgical technique under regional anesthesia of the inferior alveolar, lingual, and buccal nerves (4 mL of 2% lidocaine with norepinephrine). The incision line was carried out on the mandibular rami, distally, and in the gingival sulcus of the mandibular second molar. A release cut was then made in the oral vestibule (Cogswell incision). Using drills and elevator or Meissner forceps, the tooth was removed. After the tooth removal, the wound was revised and cleaned, and the bony margins aligned. The mucoperiosteal flap was repositioned and secured with single-knotted, non-resorbable 3–0 silk sutures. Each patient received detailed recommendations for postoperative management. Patients were advised to rinse the mouth with 0.1% chlorhexidine gluconate solution and to take 100 mg ketoprofen orally twice a day for 3 days.

**Figure 1:**
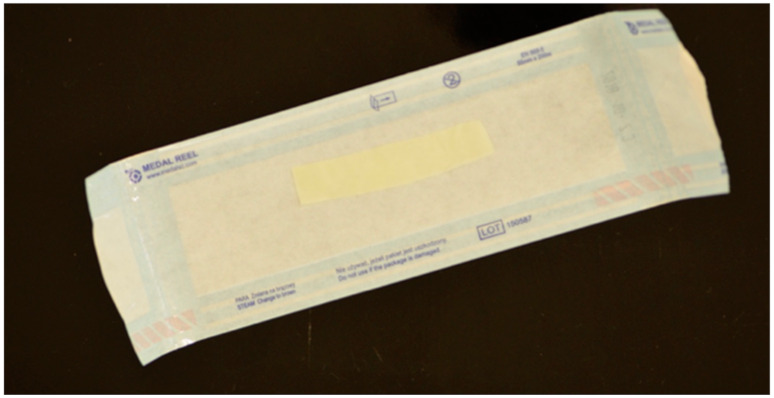
Sterile Latex flat drain.

**Figure 2:**
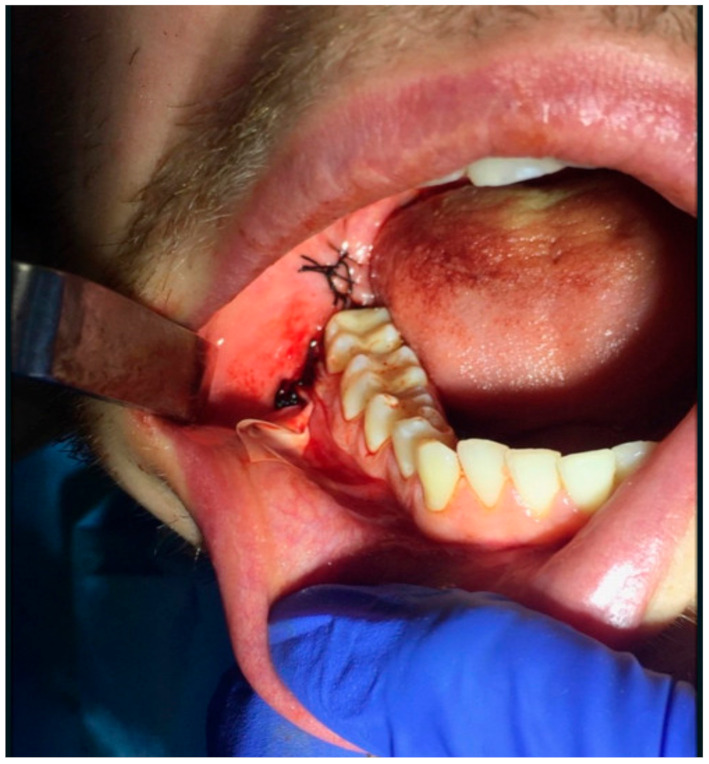
Latex flat drain sutured into a release incision in the oral vestibule.

In study group A, a sterile latex flat drain, 0.8 mm thick, 1.5–2 cm wide, and 5.5 cm long, was inserted into the floor of the oral vestibule near the releasing incision. The drain size was adjusted to the drainage needs after the removal of the lower wisdom tooth. The least possible harm to the surrounding tissues determined its width and the least potential invasiveness. Hence, a flat drain was chosen. A flat latex drain (Figure 1 and Figure 2) was inserted into the vestibular floor into the release incision below the last knot suture. The 1.5–2 cm width was not the same as, but greater than, the width of the space below the last knotted suture, resulting in slight flexion of the drain and better wound drainage without overextending the wound edges. The drain was attached with a single-knotted suture. After 24 h, at the follow-up visit, the drain was removed from the oral cavity.

**Figure 3:**
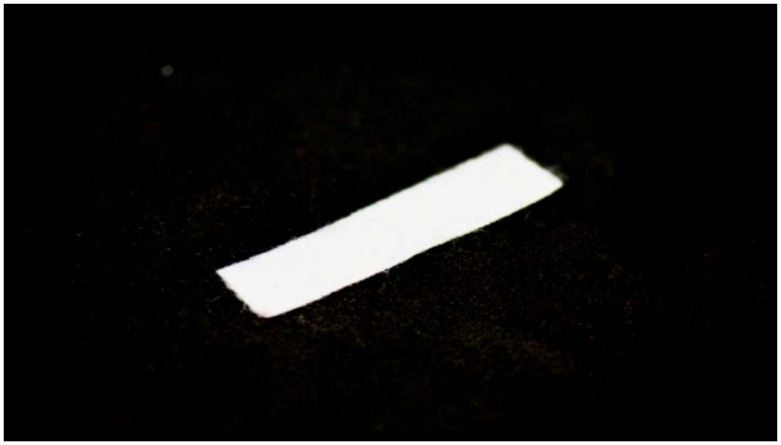
Flat Kaltostat drain.

**Figure 4:**
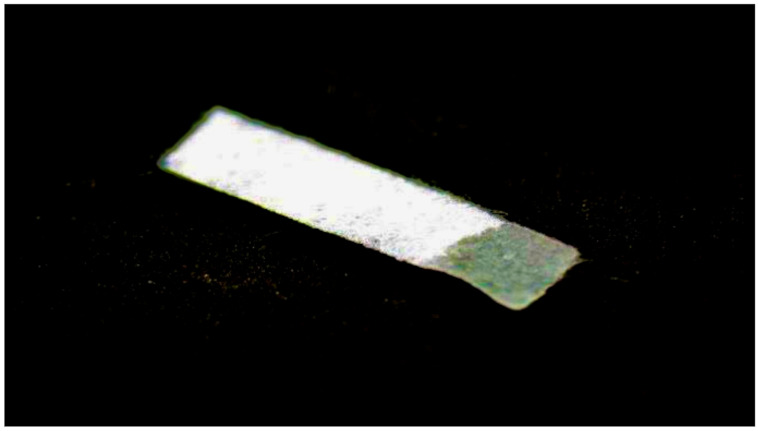
A sterile Kaltostat drain during gelation.

**Figure 5:**
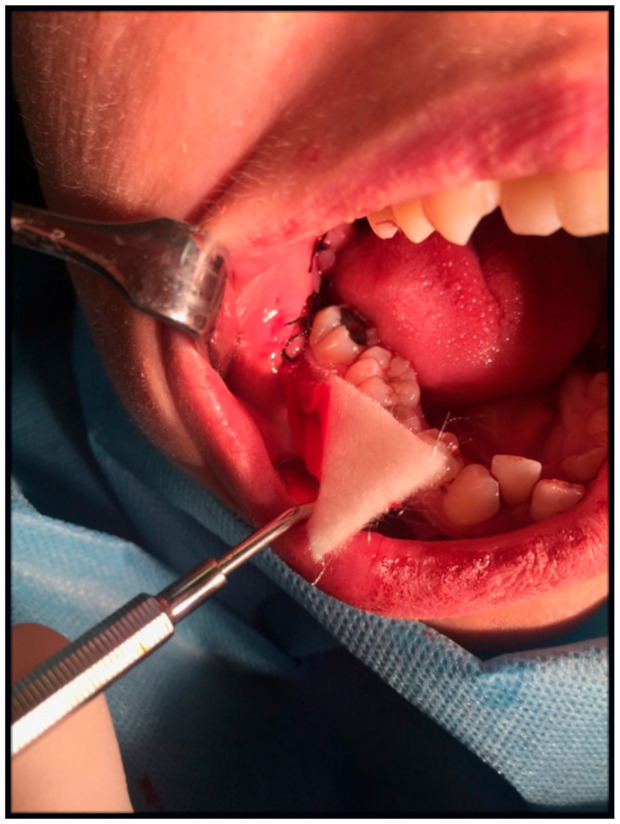
Kaltostat bud in the release incision, in the oral vestibule.

In study group B, a sterile calcium–sodium alginate drain, Kaltostat (ConvaTec Polska Sp. z o.o., Warsaw, Poland), was sutured into the atrial floor, in the region of the releasing incision, below the last knot suture (Figure 3 and Figure 4). It is a soft, nontextile, compressed white dressing recommended by the manufacturer for use after dental extractions among other things. It absorbs wound exudate, slowly turning into a gel–fibrous matrix that, by maintaining moisture and an optimal environment, does not act as a reservoir for bacteria and promotes tissue healing. In addition, it contains calcium ions, which help to accelerate the blood clotting process. The drain size was also adjusted to the extent of the operated area—factory thickness 1 mm, width 1.5–2 cm, length 5.5 cm. The drain was sewn with the same technique and in the same way as the latex drain (Figure 5). After 24 h, at the follow-up visit, it was removed from the wound.

**Figure 6:**
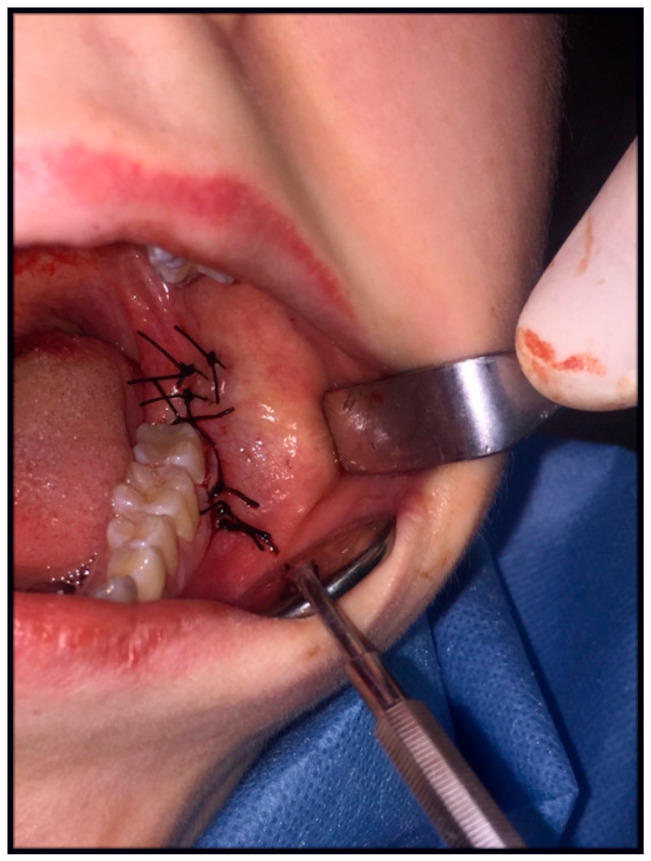
The control group—wound closure with standard knotted sutures.

Control group C consisted of patients with a wound secured by the standard method, single-knotted sutures after tooth extraction from a cut at the top of the alveolar process, and knotted sutures were placed in the oral vestibule (Figure 6).

### 2.3. Method of Assessing Postoperative Pain

**Figure 7:**
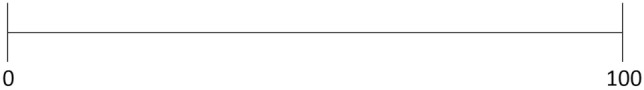
VAS scale.

The level of pain was measured using the Visual Analogue Scale (VAS). The first measurement was taken immediately before the surgical removal of the wisdom tooth. Each patient was then given the Visual Analogue Scale VAS (Figure 7), duplicated seven times. This scale ranges from 0 to 100 mm. The patient marked the perception of subjective pain in the evening on the day of surgery and the following days at the same time (12:00 pm) before taking pain medication.

### 2.4. Method for Assessing the Trismus

The measurements were made before surgery, on day 1, 2, and 7 after surgery to assess the degree of opening of the oral cavity. Using an electronic caliper, the measurement was performed in the body’s midline, between the incisal edges of the medial upper and lower incisors.

### 2.5. Method of Assessing Postoperative Soft Tissue Swelling

Each patient’s facial fixed-point analysis was performed before surgery, and the line length between the craniometric points was measured.

**Figure 8:**
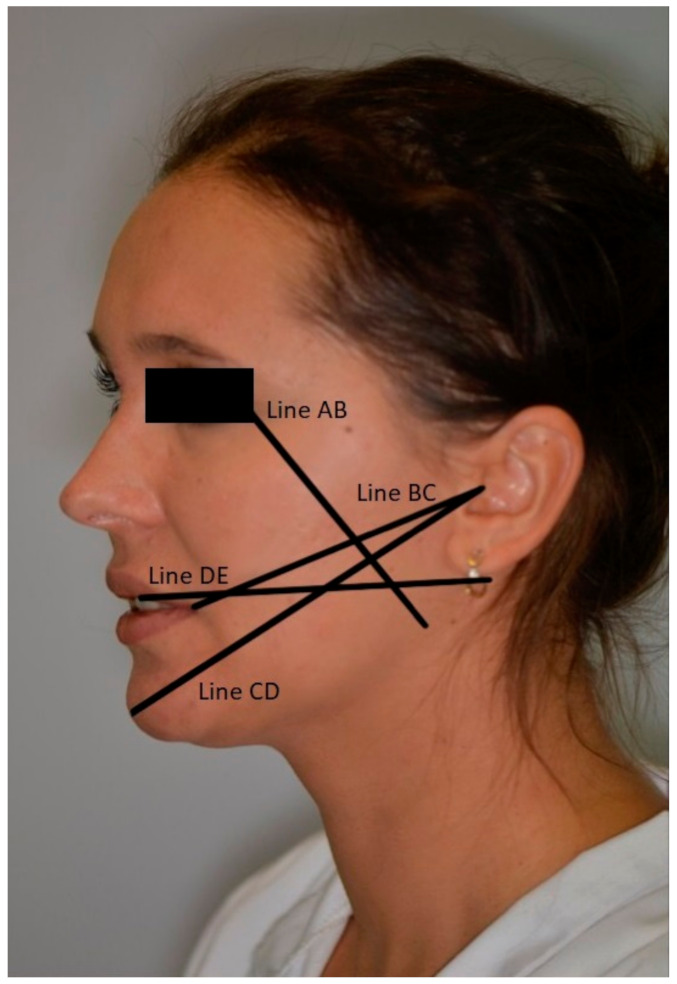
Measurement lines—AB, BC, CD, DE.

Edema was assessed using a flexible measuring technique developed by Gabka and Matsumura (Gabka and Matsumura, 1950) measuring the length of the lines connecting the corresponding craniometric points (Figure 8):

- distance between the outer angle of the eye (Exocanthion—Ex) and the angle of the mandible (Gonion—Go)—line AB;
- The distance between tragus (Tragus—T) and the angle of the mouth (Chelion—Ch)—line BC;
- The distance between the tragus (Tragus—T) and the skin point (Pogonion—WPg)—line CD;
- The distance between the medial upper incisor’s medial surface and the posterior edge of the mandibular ramus—line DE.

Measurements were taken before surgery and on day 1, 2, and 7 after surgery using an elastic measuring tape to eliminate any pressure on the facial skin.

### 2.6. Methodology of Statistical Analysis

Statistical analysis was performed with the program R, version 3.5.1. (R Foundation for Statistical Computing, Vienna, Austria, 2017). Variables were described using measures of position: quartiles—Q1, Q2 (median), Q3; arithmetic mean along with a measure of variability, i.e., standard deviation—SD.

Qualitative variables were analyzed using the chi-square test and Fisher’s exact test. Quantitative variables, when they had normal distribution, were compared using analysis of variance (ANOVA). When the distribution was not normal, the Kruskal–Wallis test was performed. Post hoc analysis was performed after detecting statistically significant differences: when distribution was normal—Fisher’s Least Significant Difference (LSD) test and when distribution was not normal—Dunn’s test. Shapiro–Wilk test was used to test the normality of the distribution of variables. The accepted significance level in statistical analysis was *p* = 0.05, so values below *p* were defined as statistically significant.

## 3. Results

### 3.1. Baseline Characteristics

Ninety patients classified for surgical removal of mandibular third molars were included in the study (*n* = 90). The patients were randomly assigned to one of three groups, each consisting of 30 patients (*n* = 30): a group that was treated with a latex flat drain sutured—referred to as group A (*n* = 30), a group that was treated with a Kaltostat sutured after surgery—referred to as group B (*n* = 30), and a group that was treated with standard knotted sutures—referred to as group C (*n* = 30). Fifty-seven women and 33 men participated in the study. The study also took into account the age of the patients, which also did not show statistically significant differences (mean 26.48 ± SD 7.03). There were no significant differences between the groups in the numbers of right and left wisdom teeth extracted. Forty-eight teeth were removed on the right side, while 42 teeth were removed on the left side of patients.

### 3.2. Analysis of the Frequency of Retention Classes According to Pell and Gregory in the Study Groups

The position and inclination of the mandibular third molars were determined using the Pell and Gregory classification. Details of the types and levels of tooth retention are shown in Table 1

### 3.3. Comparative Analysis of the Procedure Difficulty Scale According to Pederson Classification between Study Groups

The predictable difficulty of mandibular wisdom tooth removal was evaluated using the Pederson scale. Detailed results are shown in Table 2.

### 3.4. Comparative Analysis of the Level of Pain before and from Days 1 to 7 after Surgery between the Study Groups

The level of pain, determined using the VAS scale, was measured before and 7 days after surgery. All patients measured pain at zero before the procedure, i.e., none of the patients who qualified for surgical removal of wisdom teeth reported any pain before the procedure. Patients from all study groups experienced the highest level of pain on postoperative day 1. The mean value was lowest in group A at 58.33 ± 17.98 and highest in group B at 63.1 ± 24.77. There were no significant differences between groups A, B, and C in the intensity level analysis at each time point. Detailed data are summarized in Table 3.

### 3.5. Comparative Analysis of the Level of Jaw Opening before Surgery and on Day 1, 2, and 7 after Surgery between Groups

The jaw opening level was measured with a caliper between the incisor edges in the midline. There were no significant differences in the level of jaw opening between the study groups preoperatively and on days 1 and 2 (*p* > 0.05). Analysis of variance revealed that on day 7, the level of jaw opening between groups differed significantly (*p* = 0.021; ANOVA test). Significantly greater jaw opening occurred among patients in group C (39.07 ± 7.29 mm) and group A (39.6 ± 6.32 mm) compared to group B (35.05 ± 6.65). The results are summarized in Table 4.

### 3.6. Comparative Analysis of AB Line Length before Surgery and on Day 1, 2, and 7 after Surgery between Study Groups

Patients had their AB line lengths measured before surgery and on day 1, 2, and 7 after surgery. AB line lengths in each study group before surgery showed no statistically significant differences. The longest AB line measurements were recorded in group C patients, averaging 10.14 ± 1.05 cm. The Kruskal–Wallis test showed AB line length on day 2 (*p* = 0.011) and day 7 (*p* = 0.012) differed significantly between study groups. On postoperative day 2, the length of the AB line in patients in group C averaged 11.07 ± 1.08 cm and was significantly greater than in group A, where it was 10.27 ± 1.04 cm, and in group B—10.46 ± 1 cm. On postoperative day 7, the AB line was also significantly longer in group C compared to groups A and B, averaging 10.63 ± 0.82 cm. Detailed data are presented in Table 5.

### 3.7. Comparative Analysis of BC Line Length before Surger, and on Day 1, 2 and 7 after Surgery between Study Groups

BC line length was measured before surgery and on day 1, 2, and 7 after lower wisdom tooth removal. The BC line was longest in group C at all time points. The largest measurements among patients in group C were recorded on postoperative days 1 and 2, at 11.79 ± 0.86 cm and 11.77 ± 0.98 cm, respectively. The shortest postoperative measurements were recorded in group B, which averaged 11.51 ± 0.76 cm on day 1 and 11.52 ± 0.87 on day 2 postoperatively. However, comparative analysis of BC line length showed no significant difference in BC line length between groups (*p* > 0.05) before surgery and on day 1, 2, and 7 after surgery. Details are given in Table 6.

### 3.8. Comparative Analysis of CD Line Length before Surgery and on Day 1, 2, and 7 after Surgery between Study Groups

Before surgery, the mean baseline CD line length was 14.85 ± 0.97 cm in group A, 14.39 ± 0.99 cm in group B, and 14.51 ± 0.93 cm in group C. There were no significant differences in line lengths measured before treatment between the groups. Similar statistical analysis results were obtained on the remaining days, showing no statistically significant differences between the study groups. Detailed data are presented in Table 7.

### 3.9. Analysis of Changes in the Length of the DE Line before Surgery and on Day 1, 2, and 7 after Surgery between Study Groups

Statistical analysis revealed no significant differences in DE line length baseline measurements before surgical removal of the lower wisdom tooth. The mean DE line length in the patients on postoperative day 1 was 12.91 ± 1 cm in group A, 12.09 ± 0.64 cm in group B, and 13.29 ± 1.13 cm in group C. The above differences were statistically significant (*p* = 0.015, ANOVA test). Post hoc analysis showed that the line in group C was significantly longer than the measurements in group B. Similar analysis results were obtained at the next time point. On postoperative day 2, the mean DE line length was 12.91 ± 1.05 cm in group A, 12.52 ± 0.63 cm in group B, and 13.29 ± 1.13 cm in group C. Analysis of variance showed that the differences between groups were statistically significant (*p* = 0.01; ANOVA test). The DE line among patients in group C was significantly longer compared to the study group B. On postoperative day 7, there were no statistically significant differences between the study groups (*p* = 0.076; Kruskal–Wallis test)—Table 8.

## 4. Discussion

Many methods of treating noninfectious postoperative complications both locally and systemically have been analyzed in search of appropriate wound care that meets several essential criteria: it is painless, takes relatively little time, acts locally, is effective, and does not present the patient with the problem of numerous follow-up visits. Equally important are accessibility, simplicity of application, and patient comfort. From all possible drainage methods described in the literature, the least invasive one, i.e., flat drain application, was chosen in our study. In the present study, latex and alginate drains were used in a buccal release incision near the vestibule floor. A latex drain, or Kaltostat, measuring 1.5–2 cm/5 cm, was inserted into the wound, and sutures were placed on top of the mandibular alveolar region and in the release incision. The drain was removed on postoperative day 1, resulting in a higher healing rate and a scar that did not interfere with tissue function [15]. The technique of inserting a drain into the release incision has many advantages. The patient does not experience the discomfort caused by a round drain, as the flat drain is almost invisible in the mouth, and its removal is not painful. Suturing the drain into the release incision and securing the top of the mandibular alveolar region after surgery with knotted sutures is a minimally invasive method. Securing the extraction site with sutures reduces postoperative bleeding and prevents PEA (post-extraction alveolitis) by keeping the clot in the alveolus. By maintaining drainage for 24 h, evacuation of inflammatory exudate is expected and, because of faster removal of the drain, healing by rhizotomy, which includes the first 24 h [15].

This study aimed to evaluate the effect of intraoral drainage with flat latex and Kaltostat drains applied to the buccal side on the severity of complications after surgical removal of the lower wisdom tooth, and we present this method as easy, effective, and likely to find widespread use. The use of rubber drains for wound care after wisdom tooth extraction procedures has been reported in the literature [13,16,17,18]. It has been noted that the use of drainage reduces postoperative complications such as pain, swelling, and trismus [17,19,20,21,22]. Based on scientific reports, the primary wound closure technique was chosen for our study of inserting a flat drain into a release cut in the oral vestibule. This method reduces the accumulation of food debris in the wound, minimizes the risk of PEA formation, and minimizes the risk of excessive postoperative bleeding. In addition, a study by Gay-Escoda et al. [23] demonstrated that suture supply to the site after surgically removing the lower wisdom tooth and securing a single-knotted suture to the oral vestibular incision, without drainage applied, does not reduce pain, swelling, and trismus. According to some authors, the type of flap formed and the incision used do not affect the status of postoperative complications [24,25]. Thus, the effect of primary and secondary healing on the status of postoperative complications has begun to be studied. It is widely believed that an immediate healing process after tight wound care causes more pain and swelling than secondary healing [26,27,28,29]. Few are of the opinion that postoperative healing is not significantly different in terms of pain severity [30]. Primary wound healing is rapid and preferable after surgery; however, it results in more swelling, pain, and tenderness due to lack of evacuation of exudate and pressure on nerves and vessels [31]. It can also cause hematoma formation, which can become infected. However, keeping the clot in the alveolus and securing it with sutures is the basis of proper healing, as this prevents food accumulation in the wound, excessive bleeding, and PEA [13].

Secondary healing by granulation is uncomfortable for the patient because of the long duration, frequent disintegration of the clot leading to PEA, and the accumulation of food debris in the alveolus, which can cause tissue infection and a high risk of the formation of a pathological pocket behind the second mandibular molar [30]. Studies have shown that healing by omitting sutures after surgical extraction of the wisdom tooth [27,28,32], creating a triangular drainage space behind the second molar, and using a drain on top of the alveolar process results in secondary healing that is characterized by little pain and swelling [28,33].

An alternative to secondary healing appears to be primary healing using a drain in the floor of the oral vestibule to drain wound exudate.

Researchers have focused on keeping the drain in the postoperative wound for 72 h, resulting in secondary healing in the release incision [16]. For this purpose, a rubber drain with a round cross-section and a diameter of approximately 4 mm was used. Using such a drain was traumatizing to the tissues and uncomfortable for the patient because it caused a foreign body sensation in the mouth. The drain was fixed with knotted sutures. A flat latex drain was used in study by Hu et al. in 2017 [13]. Patients hardly noticed its presence in the oral cavity; moreover, they were less likely to have a recurrent infection than patients with a round drain. Long-term studies have shown that factors affecting healing quality include thorough wound cleaning, maintenance of a moist environment, and protection from infection [34]. Local factors that delay wound healing after surgical removal of wisdom teeth include inadequate drainage, edema, hematoma, local tissue anemia due to excessive tissue tension, the method of forming a full-thickness flap, and the type of healing—by primary healing or granulation [7,34,35].

In addition to using round drains and flat latex drains, calcium–sodium alginate is also used in the literature after tooth extractions. Turner listed the characteristics of an ideal dressing and defined those of an alginate dressing used for wound care, which was also used in our study [14,36]. According to the current literature, alginate is used as a dressing after extraction and as a drain placed in the cavity of dental abscesses [14,37]. Undoubted advantages of alginates include permeability to oxygen, maintenance of adequate tissue moisture, absorption of blood and exudate, protection against reinfection, adequate mechanical strength, anti-allergenicity, lack of adhesion to the wound, biodegradability. This material can be a drug carrier [38].

Using flat drains is a form of drainage that is less invasive than inserting round drains into the postoperative wound, inducing free flow of exudate; at the same time, it is an alternative to methods minimizing postoperative complications—steroid therapy, cryotherapy, laser therapy, Kinesio Taping [39,40,41,42,43].

Studies by the teams of Deliverska and Petkova and Seymour et al. found that pain is most severe in the immediate postoperative period and gradually decreases until it is completely gone [44,45]. A study by Seymour et al. reported that the degree of pain after surgical removal of mandibular molars supplied with sutures without drainage is most significant on the day of surgery and increases up to 12 h after surgery [45]. In our study, there was no difference in pain between the study groups during the first 24 h, which was confirmed by the analysis of Chukwuneke et al. [17]. They found no statistically significant difference between the control and study groups on postoperative day 1 in terms of pain intensity, which was consistent with their study. Additionally, Chukwuneke et al. examined pain complaints after day 3 [17]. After this time, the mean pain level score of the control group was significantly lower than that of the study group because there was a transient increase in pain in the rubber tube group, probably because of its irritating effect [18]. A sharp decrease was observed in the level of pain experienced after removing the rubber drain after day 3. In the author’s study, no significant reduction in pain was observed after removing the drain, suggesting that its presence did not affect the perception of postoperative discomfort.

Edema can be caused by tissue response to stretch, compression, or trauma associated with surgery. Its onset is gradual, with maximum swelling occurring within 48 h of surgery. It increases until the fourth day and completely subsides within the next seven days [46,47,48]. There are different ways to measure facial swelling: the use of the facial arch, cephalostat, ultrasound measurements, photography, and measuring the distance between craniometric points [49]. Measuring with a flexible tape measure the corresponding lines defined by the distances between the various fixed points on the face, the so-called craniometric points, is non-invasive and easy to perform [21].

Chukwuneke et al. evaluated the effect of drainage after surgical removal of mandibular third molars on facial swelling on day 1 after surgery [17]. It was significantly higher in the control group (13%) than in the study group (7%). In our study, the most significant swelling was found on day 1 in all study groups. However, it was significantly less in the latex knot groups and already decreasing from day 2, while in the control group, it persisted up to 48 h after the procedure.

Like swelling, the trismus usually peaks on the second day and subsides by the end of the first week. There is a strong correlation between postoperative pain and maxillo-facial tightness, indicating that pain may be one of the primary causes of reduced jaw dilation after lower wisdom tooth extraction [50].

In the present study, jaw opening was measured before surgery and after day 1, 2, and 7 after surgery, following the scheme given by Handa et al. [51]. Using an electronic caliper and measuring the maximum distance between the incisal edges of the medial incisors in the maxilla and the incisal edges of the medial incisors in the mandible, it was found that the least jaw opening occurred after day 1 in all study groups, which was consistent with the result of Rakprasitkul and Pairuchvej, Cerqueira et al., and Genc et al. [19,52,53].

As a result of the research conducted by our team, they proved that intraoral drainage with a flat drain after mandibular third molar removal does not significantly reduce pain, as measured by the VAS scale, or postoperative swelling, as measured by lines between craniometric points. Intraoral drainage with a latex drain after mandibular third molar removal does not significantly reduce trismus, while intraoral drainage with a calcium–sodium alginate drain increases trismus. Intraoral drainage with flat drains, especially latex drains, can make the patient’s recovery faster and less traumatic. It may result in a quicker return to activities of daily living and work duties. These results showed the significant clinical implications of our study. Intraoral drainage using flat drains, especially latex drains, can make the patient’s recovery faster and less traumatic. This method is cheap, simple, and can be performed in dental offices and oral surgery, even by a general dentist. It does not require a ready-made preparation—a flat latex drain can be obtained from a sterile latex glove by preparing a drain of the desired dimensions.

Limitations of the study include the small size of the study group. Only 90 people participated in the study. Moreover, one more group could be added to the study—round drains. Furthermore, the assessment of pain on the VAS scale is subjective. In addition, the measurements assessed in the study were not continuous but taken only at specific time points. In the future, more precise tests could also be performed to measure the level of maxillary and facial swelling, e.g., evaluation by extraoral scan and comparison of STL files [54].

## 5. Conclusions

Intraoral drainage with a flat drain after mandibular third molar removal does not significantly reduce pain, as measured by the VAS scale, or postoperative swelling, as measured by lines between craniometric points. Intraoral drainage with a latex drain after mandibular third molar removal does not significantly reduce trismus, while intraoral drainage with a calcium–sodium alginate drainage bag significantly increases trismus. Intraoral drainage with flat drains, especially latex drains, can make the patient’s recovery process faster and less traumatic. It may result in a quicker return to activities of daily living and work duties.

## Figures and Tables

**Table 1:** Results of comparative analysis of the frequency of each retention class according to Pell and Gregory classification in groups A, B, C.

		Group A (*n* = 30)	Group B (*n* = 30)	Group C (*n* = 30)	Groups A,B,C (*n* = 90)	*p*
Pell and Gregory	A1	5 (16.67%)	1 (3.33%)	5 (16.67%)	11 (12.22%)	0.184
	A2	8 (26.67%)	10 (33.33%)	6 (20.00%)	24 (26.67%)	
	A3	0 (0.00%)	1 (3.33%)	0 (0.00%)	1 (1.11%)	
	B1	5 (16.67%)	4 (13.33%)	3 (10.00%)	12 (13.33%)	
	B2	8 (26.67%)	7 (23.33%)	4 (13.33%)	19 (21.11%)	
	B3	0 (0.00%)	2 (6.67%)	1 (3.33%)	3 (3.33%)	
	C2	0 (0.00%)	1 (3.33%)	6 (20.00%)	7 (7.78%)	
	C3	4 (13.33%)	4 (13.33%)	5 (16.67%)	13 (14.44%)	

Explanations: *n*—number of patients, *p*—(*p*-value) significance level.

**Table 2:** Results of comparative analysis of the scale of procedure difficulty according to Pederson’s classification between groups A, B, C.

		Group A (*n* = 30)	Group B (*n* = 30)	Group C (*n* = 30)	Groups A,B,C (*n* = 90)	*p*
Procedure difficulty according to Pederson	slightly difficult	9 (30.00%)	6 (20.00%)	6 (20.00%)	21 (23.33%)	0.8
	moderately difficult	12 (40.00%)	16 (53.33%)	14 (46.67%)	42 (46.67%)	
	very difficult	9 (30.00%)	8 (26.67%)	10 (33.33%)	27 (30.00%)	

Explanations: *n*—number of patients, *p*—(*p*-value) significance level.

**Table 3:** Results of comparative analysis of pain intensity measured before surgery and on the following study days after surgery between groups A, B, C.

Pain Intensity		Group A (*n* = 30)	Group B (*n* = 30)	Group C (*n* = 30)	*p*
Before surgery	Mean ± SD	0 ± 0	0 ± 0	0 ± 0	1
	median	0	0	0	P
	quartile	0–0	0–0	0–0	
1. day	Mean ± SD	58.33 ± 17.98	63.1 ± 24.77	62.1 ± 26.09	0.583
	median	64.5	66.5	60.5	NP
	quartile	46.25–72	55–75.75	45.25–85.5	
2. day	Mean ± SD	48.07 ± 18.54	53.37 ± 24.2	53.8 ± 21.08	0.516
	median	52	55.5	54.5	P
	quartile	34.5–62	39.75–72	39–69	
3. day	Mean ± SD	33.8 ± 20.81	40.93 ± 22.36	45.37 ± 21.32	0.116
	median	37	41.5	48	P
	quartile	15.25–49.75	25.5–54.75	29.25–60.5	
4. day	Mean ± SD	22.7 ± 18.48	30.27 ± 23.31	31.13 ± 21.6	0.336
	median	20	24.5	30	NP
	quartile	6.75–34	14.5–48	12.5–48	
5. day	Mean ± SD	14.07 ± 11.84	24.17 ± 21.22	23.1 ± 21.02	0.211
	median	12	19	15.5	NP
	quartile	3.25–22.75	8.5–39.25	6–41	
6. day	Mean ± SD	8.53 ± 11.05	17.37 ± 18.92	15.77 ± 17.72	0.154
	Median	3.5	13	13	NP
	Quartile	0–14.5	1–28	0–24	
7. day	Mean ± SD	3.87 ± 5.34	10.47 ± 14.98	9.43 ± 13.67	0.159
	median	1	5	3.5	NP
	quartile	0–6	0–11.75	0–15	

Explanations to the table: P—normal distribution, NP—no normality of distribution, SD—standard deviation, *n*—number of patients, *p*—(*p*-value) significance level.

**Table 4:** Results of the comparative analysis of the level of jaw opening measured before surgery and on the following study days after surgery between groups A, B, C.

Jaw Opening [mm]		Group A (*n* = 30)	Group B (*n* = 30)	Group C (*n* = 30)	*p*
Before surgery	Mean ± SD	45.87 ± 5.09	45.74 ± 6.77	47.94 ± 6.83	0.277
	median	45.89	46.86	49.22	NP
	quartile	42.05–49.69	44–50	43.27–51.99	
1. day	Mean ± SD	30.79 ± 8.72	25.87 ± 8.7	29.53 ± 9.06	0.059
	median	30	24.88	28.57	NP
	quartile	23.46–34.5	20.3–31.12	24.02–34.74	
2. day	Mean ± SD	32.56 ± 8.23	27.76 ± 7.89	30.47 ± 9.58	0.101
	median	31.16	28.75	29.3	P
	quartile	28.06–37.85	21.92–32.97	24.24–34.69	
7. day	Mean ± SD	39.6 ± 6.32	35.05 ± 6.65	39.07 ± 7.29	0.021
	median	40.37	34.5	38.56	P
	quartile	36.52–43.14	31.59–39.88	33.04–44.75	A, C > B

Explanations: P—normal distribution in groups, NP—no normality of distribution, SD—standard deviation, *n*—number of patients, *p*—(*p*-value) significance level.

**Table 5:** Comparative analysis of AB line length before treatment and on day 1, 2, and 7 after treatment between groups A, B, C.

Line AB [cm]		Group A (*n* = 30)	Group B (*n* = 30)	Group C (*n* = 30)	*p*
Before surgery	Mean ± SD	9.94 ± 0.98	9.98 ± 0.93	10.14 ± 1.05	0.701
	median	10	10	10	P
	quartile	9.12–10.5	9.5–10.43	9.5–10.95	
1. day	Mean ± SD	10.32 ± 1.06	10.75 ± 1.16	11.02 ± 1.11	0.057
	median	10.25	11	11	P
	quartile	9.5–11	10–11.3	10.12–12	
2. day	Mean ± SD	10.27 ± 1.04	10.46 ± 1	11.07 ± 1.08	0.011
	Median	10	10.25	11	NP
	quartile	9.5–10.5	10–11	10–11.88	C > B, A
7. day	Mean ± SD	10.07 ± 1	10.11 ± 0.83	10.63 ± 0.82	0.012
	median	10	10	10.5	NP
	quartile	9.5–10.5	9.5–10.5	10–11	C > B, A

Explanations: P—normal distribution in groups, NP—no normality of distribution, SD—standard deviation, *n*—number of patients, *p*—(*p*-value) significance level.

**Table 6:** Results of comparative analysis of BC line length before treatment and on day 1, 2, and 7 after treatment between groups A, B, C.

Line BC [cm]		Group A (*n* = 30)	Group B (*n* = 30)	Group C (*n* = 30)	*p*
Before surgery	Mean ± SD	11.14 ± 0.73	10.84 ± 0.68	11.19 ± 0.9	0.169
	median	11.35	10.9	11	P
	quartile	10.5–11.5	10.5–11.45	10.5–11.73	
1. day	Mean ± SD	11.51 ± 0.76	11.49 ± 0.64	11.79 ± 0.86	0.239
	Median	11.5	11.5	11.6	P
	quartile	11–12	11–12	11–12.5	
2. day	Mean ± SD	11.52 ± 0.87	11.45 ± 0.64	11.77 ± 0.98	0.302
	median	11.5	11.45	11.9	P
	quartile	11–12.07	11–11.95	11–12.5	
7. day	Mean ± SD	11.28 ± 0.77	11.12 ± 0.66	11.5 ± 0.91	0.18
	median	11.45	11	11.5	P
	quartile	11–12	10.53–11.5	11–12	

Explanations: P—normal distribution, SD—standard deviation, *n*—number of patients, *p*—(*p*-value) significance level.

**Table 7:** Results of comparative analysis of CD line length before treatment and on day 1, 2, and 7 after treatment between groups A, B, C.

Line CD [cm]		Group A(*n* = 30)	Group B (*n* = 30)	Group C (*n* = 30)	*p*
Before surgery	Mean ± SD	14.85 ± 0.97	14.39 ± 0.99	14.51 ± 0.93	0.174
	median	14.5	14.45	14.5	P
	quartile	14.1–15.5	13.5–15	14–15	
1. day	Mean ± SD	15.39 ± 1.05	15.16 ± 0.9	15.26 ± 0.94	0.661
	median	15.4	15	15.1	P
	quartile	14.62–16	14.5–15.57	15–16	
2. day	Mean ± SD	15.28 ± 0.96	15.07 ± 0.87	15.19 ± 0.97	0.686
	median	15.35	15	15	P
	quartile	14.5–15.5	14.5–15.65	14.5–15.5	
7. day	Mean ± SD	15.01 ± 1.01	14.62 ± 0.97	14.77 ± 0.88	0.296
	median	15	14.5	15	P
	quartile	14.5–15.5	14–15.38	14.5–15.15	

Explanations: P—normal distribution, SD—standard deviation, *n*—number of patients, *p*—(*p*-value) significance level.

**Table 8:** Results of comparative analysis of DE line length before treatment and at day 1, 2, and 7 after treatment between groups A, B, C.

Line DE [cm]		Group A (*n* = 30)	Group B (*n* = 30)	Group C (*n* = 30)	*p*
Before surgery	Mean ± SD	12.45 ± 1	12.09 ± 0.64	12.62 ± 1.04	0.127
	median	12.5	12	12.5	NP
	quartile	12–12.95	11.5–12.5	12–13	
1. day	Mean ± SD	12.91 ± 1	12.53 ± 0.65	13.25 ± 1.11	0.015
	median	13	12.5	13	P
	quartile	12.5–13.5	12.25–13	12.5–14	C > B
2. day	Mean ± SD	12.91 ± 1.05	12.52 ± 0.63	13.29 ± 1.13	0.01
	median	12.9	12.5	13	P
	quartile	12.2–13.5	12.1–12.7	12.5–14	C > B
7. day	Mean ± SD	12.74 ± 1.04	12.29 ± 0.63	12.89 ± 1.05	0.076
	median	12.65	12.5	12.6	NP
	quartile	12–13.5	12–12.65	12.05–13.45	

Explanations: P—normal distribution in groups, NP—no normality of distribution, SD—standard deviation, *n*—number of patients, *p*—(*p*-value) significance level.

## Data Availability

Data are available on request because of privacy or ethical restrictions.
